# An Effective Way of Producing Fully Assembled Antibody in Transgenic Tobacco Plants by Linking Heavy and Light Chains via a Self-Cleaving 2A Peptide

**DOI:** 10.3389/fpls.2018.01379

**Published:** 2018-09-19

**Authors:** Yuan Lin, Chiu-Yueh Hung, Chayanika Bhattacharya, Starr Nichols, Hafsa Rahimuddin, Farooqahmed S. Kittur, TinChung Leung, Jiahua Xie

**Affiliations:** ^1^Department of Pharmaceutical Sciences, Biomanufacturing Research Institute and Technology Enterprise, North Carolina Central University, Durham, NC, United States; ^2^School of Basic Medical Sciences, Ningxia Medical University, Yinchuan, China; ^3^Department of Biological and Biomedical Sciences, Julius L. Chambers Biomedical Biotechnology Research Institute, North Carolina Central University, Durham, NC, United States

**Keywords:** 2A self-cleaving peptide, Ebola virus antibody, similar expression levels of two genes, plant-based expression system, *Nicotiana tabacum*

## Abstract

Therapeutic monoclonal antibodies (mAbs) have evolved into an important class of effective medicine in treatment of various diseases. Since the antibody molecule consists of two identical heavy chains (HC) and two light chains (LC), each chain encoded by two different genes, their expressions at similar levels are critical for efficient assembly of functional recombinant mAbs. Although the plant-based expression system has been tested to produce fully assembled recombinant mAbs, coordinately expressing HC and LC at similar levels in a transgenic plant remains a challenge. A sequence coding for a foot-and-mouth disease virus (FMDV) 2A peptide has been successfully used to link two or more genes, which enable the translated polyprotein to be “self-cleaved” into individual protein in various genetically modified organisms. In the present study, we exploited the usage of F2A in Ebola virus monoclonal antibody (EBOV mAb) production. We found that transgenic tobacco plants carrying a transcription unit containing *HC* and *LC* linked by *2A* not only produced similar levels of HC and LC but also rendered a higher yield of fully assembled EBOV mAb compared to those expressing *HC* and *LC* in two independent transcription units. Purified EBOV mAb bound to an Ebola epitope peptide with apparent *K_d_*-values of 90.13–149.2 nM, indicating its proper assembly and high affinity binding to Ebola epitope peptide. To our knowledge, this is the first report showing mAb production by overexpressing a single transcription unit consisting of *HC*, *LC* and 2A in stable transformed tobacco plants.

## Introduction

The advent of recombinant DNA technology has launched a revolution in biotechnology by enabling production of a diverse range of biopharmaceuticals in a variety of heterologous hosts. Among various expression systems, plant-based expression system is very attractive because it offers many potential advantages, such as low cost, ease of scaling up in production, and no risk of transmitting mammalian pathogens (Daniell et al., 2001; Ma et al., 2003). Many biopharmaceuticals, including monoclonal antibodies (mAbs), have been tested in this expression system, but several challenges remain (De Muynck et al., 2010; Desai et al., 2010; Xu et al., 2012; Chen et al., 2016). Unlike most recombinant proteins with a single polypeptide, a fully assembled tetrameric form of a mAb consists of two identical heavy chains (HCs) and two identical light chains (LCs), which are encoded by two separate genes. In order to efficiently produce fully assembled functional mAbs, both genes need to express at the same or similar levels (De Muynck et al., 2010; Chng et al., 2015). Although the plant-based expression system has been widely studied to produce mAbs during the past three decades (De Muynck et al., 2010; Chen et al., 2016) since the first successful case of expressing functional mAb in plants was reported in 1989 (Hiatt et al., 1989), to achieve similar expression levels of HC and LC remains one of the major challenges.

In plant genetic engineering studies, several strategies have been utilized to express two or more transgenes in the same transgenic plant, such as sexual crossing transgenic plants each carrying different transgene (Hiatt et al., 1989; Ma et al., 1995), co-transformation of transgenes sequentially or simultaneously (Chen et al., 1998; Lapierre et al., 1999), and a tandem array of multiple transgenes each one with its own promoter and terminator in the same expression vector (Halpin, 2005; De Muynck et al., 2010; Luke and Ryan, 2013). The common drawback of these strategies is a large variation of expression levels of different transgenes in the same transgenic plant (Maqbool and Christou, 1999; Halpin, 2005). To overcome this problem, expressing multiple transgenes under the control of a single promoter as a single transcript has been suggested. Several linkers have been adopted for connecting multiple transgenes, such as a protease-susceptible linker sequence (Urwin et al., 1998), an internal ribosome entry site (IRES) sequence (Urwin et al., 2000), and a sequence encoding NIa protease (Marcos and Beachy, 1994). However, none has been widely applied because of certain limitations of each technique as summarized by Halpin (2005).

Besides the above mentioned linkers, currently, the most popular type of linker is the self-cleaving 2A peptide. For example, a sequence coding for a foot-and-mouth disease virus (FMDV) self-cleaving 2A (F2A) peptide has been used to link two genes to generate a synthetic polyprotein in plants, which could produce individual functional proteins by 2A peptide undergoing self-cleaving mechanism (Halpin et al., 1999; Ralley et al., 2004; Luke et al., 2015). The F2A peptide has 19 amino acids while some other 2A peptides have 18–22 amino acids. All of them are originated from various viruses but have similar functions. They have also been tested in mammalian, human, insect, yeast and fungal cells by co-expressing functional proteins (Halpin, 2005; De Felipe et al., 2006; Luke et al., 2015). It is believed that 2A possesses a non-proteolytic self-cleaving function instead, by using a ribosomal skipping mechanism to skip the synthesis of the glycyl-prolyl peptide bond at the *C*-terminus of a 2A peptide, leading to a cleavage of the peptide bond between the last two amino acids G and P (Donnelly et al., 2001a,b; Luke and Ryan, 2013; Luke et al., 2015). The major drawback using the 2A peptide as a linker to produce a polyprotein is the resulting proteins having additional 18–21 amino acids from 2A added to the *C*-terminus of the first protein and an additional P left from 2A at the *N*-terminus of following protein (Halpin, 2005; Luke and Ryan, 2013; Luke et al., 2015). These additional amino acid(s) raise some concern about the folding and post-translational modification of certain proteins.

The self-cleaving 2A peptide has been studied in therapeutic mAb expression in mammalian cells (Fang et al., 2005; Chng et al., 2015). However, there was no report using 2A for expressing mAb in plants when we initiated this study. The current study was to investigate the utilizability of F2A to express mAb in plant-based expression system by analyzing the expression levels of HC and LC, the cleavage efficiency of produced polyprotein HC+2A+LC and assembling efficiency of mAb in transgenic tobacco plants. Toward this end, we selected a mAb targeting the surface glycoprotein of Ebola virus (EBOV). EBOV is one of the most virulent infectious pathogens to cause acute, severe and often fatal illness in humans (Bausch et al., 2008). So far, there is no approved vaccine or treatment to effectively apply to Ebola outbreak managements^1^. Fortunately, several previous reports have demonstrated that antibodies are crucial for host survival from EBOV infection (Dye et al., 2012; Wong et al., 2012; Marzi et al., 2013). Plant-based expression system together with transient expression strategy has been employed to produce EBOV mAbs (Zeitlin et al., 2011; Olinger et al., 2012). Plant-produced single type mAb and a combination of two to three types of mAbs have been reported to effectively neutralize EBOV (Zeitlin et al., 2011; Olinger et al., 2012; Qiu et al., 2014). However, low efficacy and low production levels of mAbs using plant-based expression systems limits its application.

In the present study, we transformed a synthetic gene encoding Ebola LC, 2A, and Ebola HC (*LC* + *2A* + *HC*) driven by *GapC* promoter with *GapC* terminator (*GapCP*::*HC* + *2A* + *LC::GapCT*) into tobacco plants to produce EBOV mAb. Our results showed that most of transgenic tobacco plants expressing this synthetic gene showed similar transcriptional and translational levels of *LC* and *HC* whereas transgenic plants with a genetic cassette containing separate *LC* and *HC* with its own promoter and terminator showed more variation levels. Results from transgenic plants with *GapCP*::*HC + 2A + LC::GapCT* also showed that produced LC+2A+LC polyprotein could be cleaved and assembled into fully active mAb in plant cells. Very recently, Chen and co-workers reported that 2A works efficiently to produce bioactive Bevacizumab mAb in transgenic rice callus (Chen et al., 2016). Our results are consistent with the above report, demonstrating that 2A is valuable for mAb production in plants. These fast growing transgenic tobacco plants are also useful for mass production of EBOV mAb.

## Materials and Methods

### Plant Materials

Tobacco (*Nicotiana tabacum* L.), cultivar “Wisconsin 38,” was used in the present study to generate transgenic plants. The preparation of sterilized seedlings and the procedure for transformation are the same as described previously (Musa et al., 2009). Transgenic plants were grown under greenhouse conditions. T0 transgenic plants were first used for transgene analysis and protein characterization. Harvested T1 seeds from selected T0 transgenic plants were further subjected to kanamycin resistant screening. T1 seedlings from selected lines with 3 to 1 ratio of resistant to sensitive segregation were further grown to isolate large quantities of mAbs for the Ebola epitope peptide affinity binding assay.

### Vector Construction and *Agrobacterium*-Mediated Transformation

**FIGURE 1:**
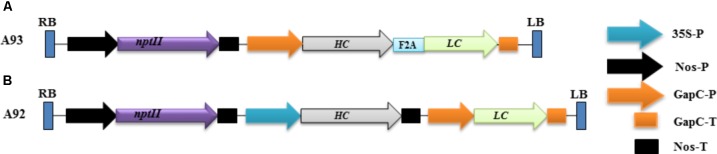
Schematic representation of the genetic cassettes A93 **(A)** and A92 **(B)**. A kanamycin resistance gene neomycin phosphotransferase (*nptII*) under the control of nopaline synthase gene promoter (nos-P) and terminator (Nos-T) was used as a selective gene in both genetic cassettes. A93 contained a DNA sequence coding for *LC* + *A2* + *HC* flanked by a glyceraldehydes-3-phosphate dehydrogease gene promoter (GapC-P) and terminator (GapC-T). A92 contained DNA sequences coding for *HC* and *LC*, which was flanked by a CaMV 35S promoter and Nos-T, GapC-P and GapC-T, respectively. RB: right border; LB, left border.

In the present study, the DNA sequences encoding HC (*HC*) and LC (*LC*) were kindly provided by Dr. Michael H. Pauly, Mapp Biopharmaceutical Inc., whose sequences were designed based on previous report by Zeitlin et al. (2011). A short DNA sequence (5′-GCCCCGGTGAAGCAGACCCTGAACTTCGACCTGCTGAAGCTGGCGGGCGACGTGGAGAGCAACCCGGGCCCC-3′) coding for F2A (Ryan et al., 1991) was used as a linker between *HC* and *LC*. Two binary vectors (**Figure 1**) were created for producing transgenic tobacco plants. The first one (designated as A93) contained a DNA sequence for *LC + A2 + HC* driven by *GapC* promoter with *GapC* terminator. The second one (designated as A92) contained *HC* and *LC* driven by *CaMV 35S* and *GapC* (Musa et al., 2009) constitutive promoters with *Nos* and *GapC* terminators, respectively. The resulting two constructs were separately introduced into *Agrobacterium tumefaciens* strain LBA4404 using freeze-thaw method (Holsters et al., 1978). An *Agrobacterium*-mediated leaf disc transformation system was used to create kanamycin resistant plants as described previously (Musa et al., 2009). A total of 17 kanamycin resistant plants from A93 and 16 from A92 were obtained for downstream analysis.

### PCR, RT-PCR, and qRT-PCR

To confirm the presence of transgenes *HC*, *LC* and *nptII* in kanamycin resistant plants, PCR amplification was performed using primer pairs H-LCF (5′-ATGGGATGGAGCTGGATCTT-3′) and HCR (5′-ATTCCTACTACTTACCAGGAGA-3′) for *HC*, and NPT-II5′ (5′-GTGGATCCCGCATGATTGAA-3′) and NPT-II3′ (5′-TCGGATCCCTCAGAAGAACT-3′) for *nptII*. For *LC*, primer pair H-LCF and LCR (5′-TGAATAGGATCCTAAGAACACTCAGTA-3′) were used to detect *LC* in A92 transgenic plants while another pair of primers LCA2F (5′-TCTAGGATCCGAGGTACCAATGGGA-3′) and LCA2R (5′-ATCCATGGGGCCCGGGTTGCTCT-3′) were used to detect *LC*+*2A* in A93 transgenic plants. One GUS transgenic plant from our previous study (Musa et al., 2009) was used as a negative control for all analysis. Genomic DNA was isolated using a DNeasy Plant Mini Kit (Qiagen, Germantown, MD, United States). PCR conditions for *LC*, *LC + 2A*, *HC* and *nptII* amplification were the same as reported previously (Musa et al., 2009) except for annealing temperatures of 54°C for both *LC* and *HC*, and 59°C for *LC*+*2A*. PCR products were resolved and revealed on a 1.5% (w/v) agarose gel.

RT-PCR was performed on transgenic plants to detect *LC, LC+2A*, and *HC* transcripts. Total RNA was isolated using RNeasy Plant Mini Kit (Qiagen, Germantown, MD, United States) and first strand cDNA synthesis was carried out as described previously (Musa et al., 2009). Primers and PCR amplification conditions were the same as described for genomic DNA PCR reactions except that cDNA was used as a template instead of genomic DNA. For qRT-PCR, the same RNA samples were used for first strand cDNA synthesis; and the PCR with a procedure described previously (Hung et al., 2010) was used. Two primer pairs were HC-qF (5′-TTCTCTTGGTACTCAAACCTACATCTG-3′) and HC-qR (5′-ACAAGTATGAGTCTTATCGCAGCTCTT-3′) for *HC* and LC-qF (5′-TCTGTGAAGCTTACCTGCACTCTT-3′) and LC-qR (5′-GTTCCATCACGTATCTAGGAGGTTTAG-3′) for *LC*. QRT-PCR was carried out using a QuantStudio^TM^ 6 Flex Real-Time PCR system (Applied Biosystems, Foster City, CA, United States). The primer pair from QuantumRNA^TM^ 18S Internal Standards kit (Ambion, Austin, TX, United States) targeting the *18S rRNA* was used as an internal control for both RT-PCR and qRT-PCR. For generating the standard curve of plasmid DNA, cycle threshold (*C*_t_) values with 1,000, 200, 40, and 8 picogram of plasmid DNA were plotted. For converting the sample *C*_t_-value to picogram of each equivalent plasmid DNA, Ct of each sample was first adjusted by its 18S internal control, then the adjusted Ct was calculated against the standard curve of plasmid DNA of each own genetic cassette.

### Isolation of Plant Proteins

The protocol for plant protein extraction was adopted from Chen et al. (2016). Young leaves of transgenic plants grown in greenhouse were harvested and ground into fine powder in liquid nitrogen. Each 100 mg of fine powder were mixed with 500 μL of extraction buffer (200 mM Tris–HCl pH 8, 100 mM NaCl, 400 mM sucrose, 10 mM EDTA, 1 mM PMSF and 0.05% (v/v) Tween 20), and the mixture was incubated on ice for 10 min before centrifugation in 4°C at 20,000 ×*g* for 15 min. The clear protein extract was subjected to SDS–PAGE under reducing or non-reducing conditions. The same protein isolation procedure was used for further purification by magnetic beads-based Protein A/G affinity binding (Pierce Biotechnology, Rockford, IL, United States). For quantifying the protein concentrations in crude leaf extracts, the Bradford protein assay (Bio-Rad, Hercules, CA, United States) was used.

### Purification of EBOV mAb

To purify EBOV mAb from plant protein extracts, Protein A/G Magnetic Beads were used for affinity binding. For small scale purification, beads were placed in a microcentrifuge tube, washed twice with TBST [20 mM Tris–HCl pH 7.5, 150 mM NaCl and 0.05% (v/v) Tween 20], and then each 0.25 mg of beads were incubated once with 600 μL of plant extracts (1.25 mg/mL of total proteins) at 4°C for 2 h followed by second incubation with fresh 600 μL of plant extracts overnight. Bound beads were then washed three times with TBST and ready for elution. For processing large volume of protein extract, it was incubated on ice for 30 min, filtrated through a two-layered cotton cloth and added to Protein A/G Magnetic Beads prewashed as described above. After incubation at 4°C for 16 h, a metal bar was used to separate the beads from solution. To elute the bound antibody for SDS–PAGE analysis, NuPAGE^®^ LDS sample buffer with or without reducing agent (500 mM DTT) were added to the bound beads and heated for 10 min at 70°C. To elute the bound antibody for affinity binding assay by ELISA, 100 μL of elution buffer (0.1 M glycine pH 2) was added to beads and incubated at 25°C for 10 min. The magnetic beads were removed from antibody samples using a magnetic stand. The elute was neutralized by adding 1/10 volume of 1 M Tris–HCl pH 8 and stabilized by adding BSA to 0.1% (w/v).

### Immunoblotting Analysis

For SDS–PAGE under non-reducing conditions, protein extracts were heated with NuPAGE^®^ LDS sample buffer without reducing agent at 70°C for 10 min. Protein were separated on a NuPAGE^®^ 4–12% Bis-Tris gel in NuPAGE^®^ MES SDS running buffer without antioxidant at 200 volts for 70 min. Following the separation, proteins were transferred onto a 0.2 μm pore size of PVDF membrane (Bio-Rad, Hercules, CA, United States) using NuPAGE^®^ transfer buffer without antioxidant. For SDS–PAGE under reducing conditions, protein extracts were heated with NuPAGE^®^ LDS sample buffer containing 25 or 75 mM DTT and the antioxidant were included in both running and transfer buffers as manufacturer instructed. After transferring for 110 min at 25 volts constant, the membrane was blocked with 3% (w/v) BSA dissolved in PBST at 4°C for overnight. It was then incubated at 25°C for 2 h with 1:5,000 diluted HRP-conjugated anti-human IgG (H + L) (catalog number SA00001-17, Proteintech Group, Rosemont, IL, United States) or 1:1,000 diluted HRP-conjugated anti-human lambda LC (catalog number STAR129P, Bio-Rad, Hercules, CA, United States) in blocking buffer. The luminescent signals were generated after incubation with SuperSignal^®^ West Pico Chemiluminescent substrate (Pierce Biotechnology, Rockford, IL, United States) and captured by Kodak Biomax X-ray film (PerkinElmer, Waltham, MA, United States). For visualization of the proteins separated on gel, SimplyBlue^TM^ (Invitrogen, Carlsbad, CA, United States) was used according to the manufacture instructions. For staining the PVDF membrane, 0.2% (w/v) Amido Black 10B (MP Biomedicals, Santa Ana, CA, United States) in 10% (v/v) acidic acid was used.

### Quantification of mAbs by ELISA

For quantifying the accumulated mAbs in A92 and A93 lines, the Protein A/G coated ELISA plates (Alpha Diagnostic International Inc., San Antonio, TX, United States) were used. Leaf tissues with known amount of weight were homogenized in TBST [50 mM Tris–HCl pH 8, 150 mM NaCl and 0.05% (v/v) Tween 20] with plant protease inhibitors (P9599, Sigma-Aldrich, St. Louis, MO, United States). After centrifugation at 20,000 ×*g* for 10 min at 4°C, the supernatant was collected and directly loaded to wells. The human IgG was also assayed for generating a standard curve at a range of 25, 12.5, 6.25, 3.125, 1.56, and 0.78 ng, and the extracts from GUS control plants were used as a blank for background levels. The loaded plates were incubated at 4°C for 16 h. After three-time washing with TBST, 5% fetal bovine serum was used for blocking the unoccupied protein G by incubating for 1 h at 25°C. After three-time washing with TBST, the plates were incubated with HRP-conjugated goat anti-human IgG (H + L) (Santa Cruz Biotechnology Inc., Dallas, TX, United States) for 1 h at 25°C. For colorimetric detection, ABTS [2,2′-azinobis-(3- ethylbenzthiazoline-6-sulfonate) (KPL Inc., Gaithersburg, MD, United States) was added as a substrate for peroxidase, and ABTS Peroxidase Stop Solution was used for stopping the reactions. The absorbance was measured at 405 nm under the SpectraMax M5 (Molecular Devices LLC, San Jose, CA, United States). Three batches of samples were assayed as three biological repeats. In each batch assay, two technique repeats were used.

### Affinity Assay by ELISA

For affinity assay by ELISA, the concentrations of eluted mAbs were determined by protein A/G affinity assay using protein A/G magnetic bead (Pierce) and human IgG as a standard (Pierce Biotechnology, Rockford, IL, United States). A Ebola epitope peptide (ATQVEQHHRRTDNDSTA), 17-amino acids corresponding to 401–417 region of the Zaire Ebola virus Mayinga strain glycoprotein (GP) (GenBank accession number: AAC54887), was synthesized and biotinylated at the *N*-terminus with a six carbon linear aminohexanoic (Ahx) linker (Genscript, Piscataway, NJ, United States) for the binding assay. This peptide has exhibited competitive binding ability to a EBOV mAb (clone 13F6) with Ebola membrane-anchored glycoprotein (GP), which has proven to protect BALB/c mice from a lethal challenge with mouse-adapted Ebola Zaire virus when 100 mg of purified EBOV mAbs was administered 24 h before challenge (Wilson et al., 2000). An indirect ELISA method previously reported by Beatty et al. (1987), which can be used not only to characterize the antibody for antigen detection but also to provide an estimate of dissociation constant (K_d_), was adopted to determine the affinity of plant-produced EBOV mAbs for Ebola epitope peptides.

The ELISA-based assay was performed at 25°C. The biotinylated Ebola epitope peptide (MW 2,305.5) was first immobilized onto streptavidin magnetic beads (Pierce Biotechnology, Rockford, IL, United States). Briefly, 20 μL streptavidin conjugated magnetic beads (10 mg/mL) were first blocked with 50 μL TBST with 0.5% (w/v) BSA for 30 min. Then biotinylated Ebola epitope peptide with different amounts: 0, 0.2, 1, 5, 25, 125, 625, or 1,000 ng, was added to the beads in a final volume of 80 μL and incubated for 1 h. The beads were then washed twice with 200 μL TBST containing 0.5% (w/v) BSA at 25°C for 30 min. The wash solution was removed using a magnetic stand, and the immobilized biotinylated Ebola epitope peptide/streptavidin bead complex was incubated with 100 μL TBST-sera (10% goat serum, 2% sheep serum and 0.2% (w/v) BSA) for 1 h to block any non-specific binding site for antibody. The biotinylated Ebola peptide/beads were resuspended in 25 μL TBST-sera and incubated with 5 μL of purified Ebola mAb from A93 transgenic plants at 25°C for 1 h on rocking platform. The mAb bound to biotinylated Ebola peptide-beads were washed twice with 200 μL TBST-sera for 30 min, and then blocked for additional 30 min with TBST-sera. To detect the bound Ebola antibody, the mAb bound bead complex was incubated with 1:500 dilution of HRP-conjugated goat anti-human IgG, F(ab)′2 Fragment (Life Technologies, Thermo Scientific, Waltham, MA, United States) in TBST-sera for 1 h. After washing twice with 200 μL TBST-sera for 30 min each, it was resuspended in 50 μL TBST. To develop the color and quantify the amount of bound antibody, TMB (3,3′,5,5′-tetramethylbenzidine) substrate (Pierce Biotechnology, Rockford, IL, United States) was added. The blue color was measured at 450 nm. The background was measured at 550 nm. Three repeats were used. GraphPad Prism 7 (GraphPad Software, La Jolla, CA, United States) was used for plotting and calculating Bmax and K_d_ as well as statistic best fit value of R square and standard deviation of estimation (Sy.x).

## Resulsts and Discussion

### Creation of Two Genetic Cassettes

The rational design of a genetic cassette using 2A as a linker was based on previous reports that a polyprotein consisting two proteins encoded by two DNA sequences linked by a sequence coding for 2A could be efficiently expressed and undergo complete cleavage (Halpin et al., 1999; Ralley et al., 2004; Luke et al., 2015). We therefore, hypothesized that co-expressing *LC* and *HC* linked by *2A* under one promoter in transgenic tobacco plants to produce them as a single polypeptide could increase the chance of comparable expression levels of LC and HC, which could benefit the assembly of complete antibodies. To test this possibility, a genetic construct A93 (**Figure 1A**) containing a sequence for *LC + 2A + HC* driven by *GapC* promoter and terminated by *GapC* terminator (*GapCP*::*LC + 2A + HC*::*GapCT*) was created. Another genetic construct A92 (**Figure 1B**) containing *LC* and *HC* sequences driven by tobacco *GapC* and Cauliflower mosaic virus *CaMV 35S* promoters, respectively, was created for comparison studies.

### PCR Confirmation of Transgenes in Kanamycin-Resistant Plants

The two genetic cassettes, A93 and A92, were stably transformed into tobacco plants, respectively using an *Agrobacterium*-mediated transformation. After co-culture and selection, one kanamycin-resistant shoot per leaf disc was isolated for shoot elongation and rooting so that each transformant was derived from an independent transformation event. A total of 17 kanamycin-resistant plants from A93 and 16 from A92 were rooted and screened for the presence of transgenes by PCR. One previously created transgenic plant expressing GUS (Musa et al., 2009) was included as a vector control in PCR analysis.

**FIGURE 2:**
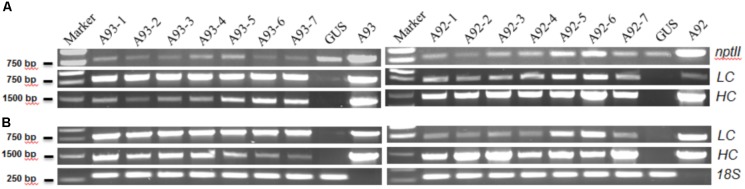
Representative results of PCR and RT-PCR analysis of A93 and A92 transgenic tobacco plants. Genomic DNAs **(A)** and total RNA-derived cDNAs **(B)** of kanamycin resistant plants were used for PCR amplification with gene specific primers. *nptII*, neomycin phosphotransferase II; *LC*, light chain; *HC*, heavy chain; *18S*, 18S rRNA gene as loading control; A93 and A92, plasmid DNA controls.

The results showed that all 17 kanamycin resistant plants from A93 transformation, 15 out of 16 from A92 and the GUS control plant had an expected PCR product of ∼820 bp amplified by NPT-II5′/3′ primers (**Figure 2A**). Similarly, these *nptII* positive A93 and A92 transgenic plants also had ∼1.41 kb PCR products of *HC* amplified by primers H-LCF/HCR while the GUS plant did not have any detectable *HC* PCR product (**Figure 2A**). To determine the presence of *LC* and *2A* in A93 kanamycin-resistant lines, primers LCA2F/LCA2R targeting *LC* + *2A* were used while primers H-LCF/LCR targeting only *LC* was used for A92 kanamycin-resistant lines. Results showed that all 17 A93 *nptII* positive lines had *LC* + *2A* PCR products of ∼800 bp while GUS plant did not. All 15 A92 *nptII* positive lines had *LC* PCR products of ∼730 bp, but GUS and one *nptII* negative A92 plants detected none (**Figure 2A**). These genomic PCR results together indicated that all 17 kanamycin-resistant plants from A93 and 15 from A92 were true transgenic plants.

### Transcription Levels of Transgenes in Transgenic Tobacco Plants

To determine whether all integrated transgenes in transgenic plants could be transcribed or not, seven confirmed transgenic plants from each of A93 and A92 were used to isolate RNA for RT-PCR to detect *HC*, and *LC* or *LC* + *2A* transcripts. The results show that all 14 plants had detectable levels of *HC* and *LC* or *LC* + *2A* transcripts (**Figure 2B**), indicating that both *HC* and *LC* transgenes in transgenic tobacco plants from two different genetic cassettes could be transcribed. However, their expression levels were not equal. In A93, four out of seven had similar transcript levels of *LC* and *HC* while remaining three had higher transcript levels of *LC* than *HC*. In A92, only two transgenic plants had similar transcript levels of *LC* and *HC* while remaining five had higher *HC* transcripts than *LC* (**Figure 2B**). This high percentage (71%) of unequal expressions of LC and HC could be partially caused by differential PCR amplification efficiencies for *LC* and *HC*. However, under the same PCR conditions, only 43% of A93 showed unequal expressions, indicating the *LC + 2A + HC* driven by *GapC* promoter and terminated by *GapC* terminator had a higher chance of producing comparable expressions of *LC* and *HC*.

In order to quantify the transcript levels of *HC* and *LC* in A92 and A93 for a better comparison, qRT-PCR was performed. To detect *HC* transcripts, the primer pair HC-qF/HC-qR was used. To detect *LC* transcripts, the primer pair LC-qF/LC-qR was used. To overcome the different amplification efficiency between *HC* and *LC*, qRT-PCR results of all A92 and A93 lines were compared to that of each plasmid DNA construct instead of optimizing the PCR conditions of *HC* and *LC* for equal amplification efficiency. The PCR amplifications of *HC* and *LC* in both plasmid DNAs as expected were very similar (**Supplementary Figure 1A**). Using A92 and A93 PCR results against standard curves of plasmid DNAs shown in **Supplementary Figure 1B** upper panel, all seven A93 lines showed similar transcript levels of *HC* and *LC* which *HC* to *LC* ratio was 0.82 ± 0.12 (*n* = 7), whereas the A92 showed much higher levels of *HC* than *LC*, except the A92-7 (**Supplementary Figure 1B**, lower panel). The average *HC* to *LC* ratio of A92-1 to A92-6 was 52.70 ± 17.28 (*n* = 6). In addition, the *LC* transcript levels in all A92 lines in average were approximately eightfold less than that in A93 lines.

### Characterization of EBOV mAb Produced in Transgenic Tobacco Plants

**FIGURE 3:**
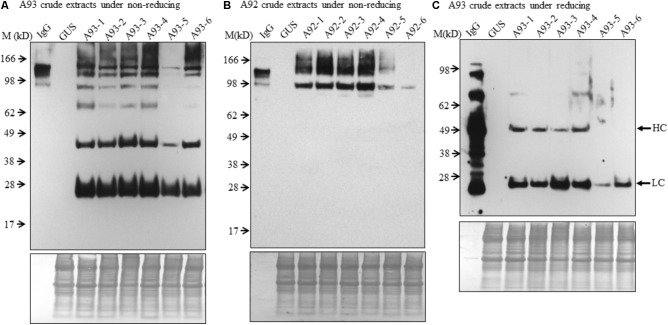
Immunoblotting analysis of crude protein extracts of A93 and A92 transgenic tobacco plants. Equal amounts of proteins from A93 **(A)** and A92 **(B)** were analyzed on SDS–PAGE under non-reducing conditions using anti-human IgG antibody. Same crude protein extracts of A93 were analyzed under reducing conditions **(C)** showed immunoreactive bands of LC at 25 kD and HC at 50 kD using anti-human IgG antibody. None was observed from A92 samples (**Supplementary Figure 2**). Bottom panels show equal protein loading by staining the blots with Amido Black 10B. IgG, 1 ng of human IgG; GUS, crude protein extracts of control plants carrying *GUS*; M, marker.

To examine the presence of assembled antibody in transgenic plants, six transgenic plants from each A93 and A92 together with a GUS control line were analyzed by immunoblotting. We first examined antibody in crude leaf extracts. Under non-reducing conditions, all six transgenic lines from A93 showed a doublet band with a size ∼150 kD which was also observed in human IgG (1 ng) positive control while GUS control plant did not show any detectable band (**Figure 3A**). This doublet band with sizes of ∼150 kD and also shown in human IgG has the calculated molecular mass equivalent to a fully assembled antibody. The doublet could be results from different sized glycan chains attached to mAb because fragment crystallizable (Fc) of IgG carries a *N*-glycan chain (van de Bovenkamp et al., 2016), and plant-produced glycoproteins including antibodies are known to bear different sizes of *N*-glycan chains resulting into different glycoforms (Jez et al., 2013; Kittur et al., 2013). It is also possible that some doublets contained incompletely assembled mAbs with two HC and one LC. Considering the immunoreactive band intensity among transgenic plants, five out of six had similar expression levels except plant A93-5, which had much less expression levels. Besides the assembled ∼150 kD doublet band, there were four other immunoreactive bands with the sizes around 100, 75, 50, and 25 kD. These bands had also been observed in transgenic tobacco plants expressing human IgG_1_ antibody (Hehle et al., 2011). According to previous report by Hehle et al. (2011), it can be reasonable to postulate that 100 kD band could correspond to a dimer of two HC while 75 kD band could result from one HC and one LC, or LC trimer. As for observed 50 and 25 kD bands, they could be some un-assembled free HC (50 kD) and LC (25 kD). Unlike the A93, all A92 transgenic plants exhibited higher molecular weight bands even larger than those observed in IgG positive control (**Figure 3B**). Besides these, there was also a 100 kD band, which could be HC dimers. However, no 50 or 25 kD band was observed.

To understand the variation in expression levels of HC and LC in A93 and A92 transgenic plants, crude protein extracts were examined under reducing conditions with 25 mM DTT and protein levels of HC and LC were analyzed by immunoblotting. Results showed that a ∼25 kD LC band was observed in all six A93 transgenic plants and a ∼50 kD HC band was detected in four of them (**Figure 3C**). On the other hand, neither 25 nor 50 kD band was detected in any of the six A92 transgenic plants except positive control (**Supplementary Figure 2**) despite that the experiment was repeated several times. Since both A93 and A92 crude extracted samples were loaded in equal amount and the same immunoblotting analysis was performed using the same antibody, no detection of LC or HC under reducing conditions in A92 was inconsistent with the results observed under non-reducing conditions (**Figure 3B**).

**FIGURE 4:**
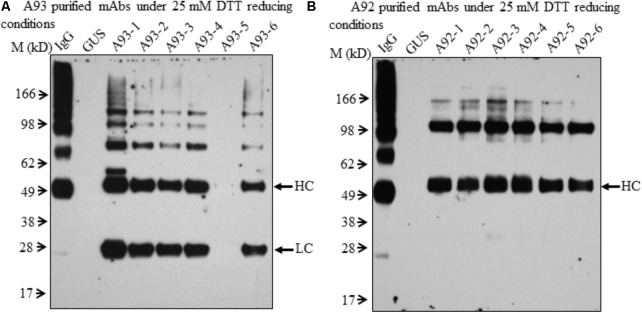
Immunoblotting analysis of purified mAbs isolated from A93 and A92 transgenic tobacco plants. Equal amounts of purified mAbs from A93 **(A)** and A92 **(B)** were analyzed on SDS–PAGE under reducing conditions with 25 mM DTT. Using anti-human IgG antibody, immunoreactive bands of LC (25 kD) and HC (50 kD) were observed in A93 but only HC was observed in A92. IgG, 1 ng of human IgG; GUS, crude protein extracts of control plants carrying *GUS*.

In order to further examine the presence of LC and HC under the reducing conditions in A92 transgenic plants, enriched mAb was analyzed. We used Protein A/G Magnetic Beads to bind the plant-produced EBOV mAb using 10-fold increased volume of crude leaf extracts. Then purified EBOV mAb was subjected to immunoblotting analysis. Under the reducing condition in the blot containing 25 mM DTT, mAb from A93 showed similar results as observed in crude extracts (**Figure 3C**) in which both LC and HC were detected with similar levels in five transgenic plants except A93-5 (**Figure 4A**). In addition to the LC and HC monomers, three bands of sizes ∼75, 100, and 150 kD were also detected (**Figure 4A**), which could represent partially assembled and intact IgG HC and LC combinations under current mild reducing conditions with 25 mM DTT. However, using the same purification and detection methods, purified mAbs from A92 transgenic plants revealed only 50 kD HC band, but no 25 kD LC consistently in all six transgenic plants (**Figure 4B**). Moreover, a 100 kD was detected which could be a dimer of HC, and a ∼150 kD band possibly corresponding to HC trimer as postulated by Hehle et al. (2011) was detected. These results indicate that LC did not accumulate or accumulated less in A92 transgenic plants. The less LC present in A92 lines than that in A93 lines was in a good agreement with the qRT-PCR result (**Supplementary Figure 1B**). Although A92-4 had the highest *LC* transcript level close to that of A93-5, its LC probably would be too little to be detected as observed A93-5 (**Figures 3C**, **4A**).

**FIGURE 5:**
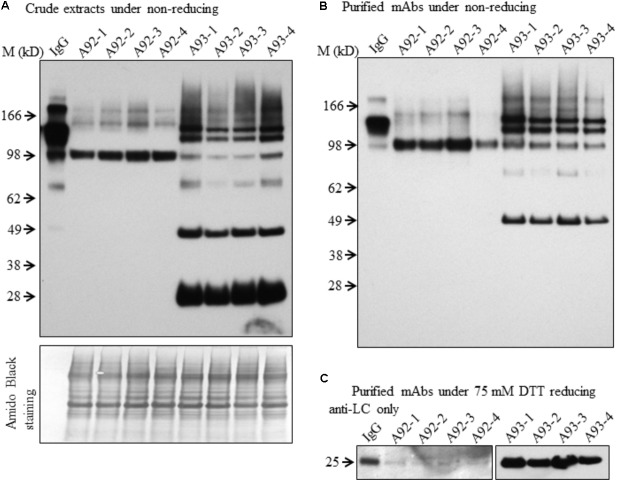
Immunoblotting comparison of mAbs isolated from selected A93 and A92 transgenic tobacco plants. Equal amount of crude protein extracts **(A)** or purified mAbs **(B)** from four A92 and four A93 lines were analyzed on SDS–PAGE under non-reducing conditions. Anti-human IgG detected doublet immunoreactive bands of fully assembled mAbs around 150 kD. **(C)** Equal amounts of purified mAbs were also analyzed under elevated reducing condition with 75 mM DTT using anti-human λLC antibody. A weaker LC at 25 kD was detected in A92 while the same band was easily detected in A93. IgG, 1 ng human IgG in **(A,B)**, while 0.5 ng in **(C)**.

Although both A93 and A92 under non-reducing conditions revealed a larger doublet band of approximated 150 kD (**Figures 3A,B**) possibly related to fully assembled antibody, to better compare the levels of fully assembled mAbs between A93 and A92, protein A/G ELISA was employed using leaf crude extracts (**Supplementary Figure 3**). The results showed that in average A93 lines have about twofold higher mAbs than A92 lines, ranging from 3 to 17 ng per mg of total soluble proteins (TSP) in A93 while 2–7.5 ng per mg of TSP in A92. According to their mAbs levels, four samples having the most abundant expressions from each A92 and A93 were selected and analyzed side by side. First, proteins from crude extracts were used. Under non-reducing condition, we found that the two large bands in A92 had larger molecular size (>150 kD) than the doublets observed in A93 and human IgG positive control (**Figure 5A**). The most abundant band in A92 was ∼100 kD, which is likely a dimer of HC. The same size band was also present in A93 transgenic plant extracts, but was at considerably lower levels. Next, protein A/G magnetic bead-based purification was carried out to enrich mAb produced from each selected transgenic plant for further comparison. When purified antibodies were used, the doublet band of ∼150 kD (**Figure 5B**) were again detected in both A93 and human IgG positive control but not in A92, indicating that A93 had much more fully assembled antibody than A92. Besides this doublet band corresponding to fully assembled antibody, there were additional immunoreactive bands with approximated sizes of 100, 75, and 50 kD in A93 plants (**Figures 5A,B**). In the case of A92 transgenic plant extracts, only a 100 kD band was present (**Figures 5A,B**). Detection of 100 kD and lower molecular weight fragments on immunoblots suggests that the protein A/G magnetic beads used for mAb purification not only bind fully assembled antibody but also could bind to the Fc (fragment crystallizable) region of HC (Rodrigo et al., 2015; Choe et al., 2016). This might contribute some to overestimated mAb levels in A92 using protein A/G ELISA (**Supplementary Figure 3**). Because the Fc region is not present in LC, LC band was not observed when protein A/G purified mAbs were analyzed under non-reducing conditions (**Figure 5B**).

Since no LC was ever detected in A92 using the anti-human IgG (H+L) (Proteintech Group) under both non-reducing and mild reducing conditions, enriched and purified antibodies were analyzed under stronger reducing condition (75 mM DTT) for increasing the levels of LC disassociating from assembled mAb or other combinations of mAb fragment complexes. In addition, to pinpoint specifically the LC, the antibody used for detection was raised against the LC only. The result confirmed that the levels of LC in A92 mAb were dramatically lower, at almost undetectable level, than those in A93 such that the same blot was exposed only a few seconds for showing LC band in A93 samples and hours for that in A92 samples (**Figure 5C**).

Based on above results, we concluded that the HC levels in A92 transgenic plants were dramatically higher than those of LC. It is known that the ratio of HC and LC is critical for folding and assembly of mAbs (Ho et al., 2013; Chen et al., 2016). For mAb engineering in mammalian cells, it has been reported that slightly high levels of LC favor the high yield of fully assembled mAbs (Schlatter et al., 2005; Ho et al., 2012). Hence, low level accumulation of LC might have resulted in less assembled mAb present in A92 compared to A93.

### Binding Efficiency of Plant-Produced EBOV mAb to a Ebola Epitope Peptide

**FIGURE 6:**
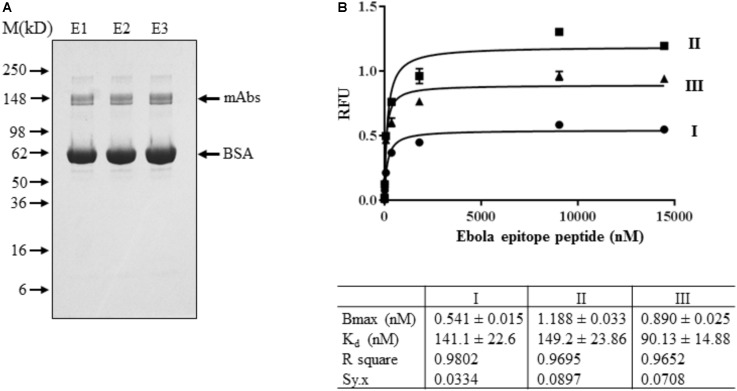
Binding affinity assay of mAbs isolated from A93 T1 transgenic tobacco plants. **(A)** Purified mAbs sequentially eluted (E1-E3) from protein A/G magnetic beads were analyzed on SDS–PAGE under non-reducing conditions. SimplyBlue SafeStain kit revealed a fully assembled mAb around 150 kD. BSA was added in purified samples for stabilizing the mAbs. **(B)** For binding affinity assay, ∼2.5 ng of purified mAbs from E1 was used for each concentration of Ebola epitope peptide from 0 to 14,461 nM. Data plotted was the results of three biological assays.

EBOV mAb purified from T1 transgenic plants was used for binding efficiency study. Using T1 transgenic plants also allow us to check the transgene stability of *GapCP*::*LC + 2A + HC*::*GapCT* in host plants. Therefore, seeds from fourteen A93 T0 transgenic plants were harvested and their kanamycin-resistant segregations were tested. Four T0 plants clearly exhibited 3 to 1 ratio of resistant to sensitive segregation. Four and five kanamycin-resistant T1 plants from A93-1 and A93-2 line, respectively, with 3 to 1 segregation were planted (**Supplementary Figure 4**) and about 35 g of leaves were pooled for isolating mAbs. Using the Protein A/G Magnetic Beads (Pierce) purification method, about 2–3 μg of purified mAbs were obtained. Protein concentration was determined by comparing its immunoreactivity with known amount of human IgG (**Supplementary Figure 5**). Using the protein A/G ELISA for quantification, about 56 ng of mAbs per mg of TSP was found to be present in leaf crude extracts which was approximately fivefold higher than that in selected two T0 plants (**Supplementary Figure 3**). Based on the 3:1 ratio of μL extraction buffers used per mg of tissues and the yield of protein concentrations at ∼2 mg/mL, it should have about 12 μg of mAbs produced in 35 g of A93 T1 leaves, indicating that an improved mAb isolation method is needed. Nevertheless, the antibody purity revealed on gel under non-reducing condition showed only the doublet band around 150 kD in Coomassie SimplyBlue stain (Invitrogen, Carlsbad, CA, United States) (**Figure 6A**), suggesting that genetic cassette *GapCP*::*LC + 2A + HC*::*GapCT* is stable in T1 transgenic plants to produce fully assembled mAbs.

Ebola epitope of the competition group 1 was derived from the membrane-anchored glycoprotein (GP), which is the viral protein known to be present on both surfaces of virons and infected cells (Wilson et al., 2000). This 17-amino acid peptide could compete the binding of GP to Ebola antibody (clone 13F6) for neutralization (Wilson et al., 2000). Therefore, it was used to characterize engineered tobacco plant-produced mAbs. Our indirect ELISA assay showed that plant-produced EBOV mAbs could detect Ebola epitope peptide with a wide range of concentrations from ∼2 to 15,000 nM (**Figure 6B**), which indicates that EBOV mAbs purified from A93 transgenic plants are functional. The apparent K_d_ was found to be the range of 90.13–149.2 nM, which is within the normal ranges of K_d_ described for antigen and antibody interactions (Kuo and Lauffenburger, 1993).

## Conclusion

In conclusion, A93 transgenic plants carrying a genetic cassette with *HC* and *LC* linked by *2A* (**Figure 1A**) not only produced similar levels of HC and LC (**Figure 4A**) but also efficiently produced fully assembled EBOV mAbs (**Figures 3A**, 5A,B). In contrast, A92 transgenic plants carrying *HC* and *LC* driven by different promoters and terminators (**Figure 1B**) could not produce enough LC (**Figures 4B**, **5C**) in comparison with HC (**Figure 4B**). The cause of low LC production in A92 is not clear, but its low protein expression resulted in low level of assembled mAbs in A92 compared to A93 (**Figures 5A,B**). Overall, cassette A93 is better than A92 for antibody production and 2A self-cleaving peptide can efficiently produce polypeptide HC+2A+LC into functional HC and LC which facilitates antibody assembly. In addition, our results also indicate that this 2A strategy linking multicistronic sequences could be stably transferred to T1 generation. With the expected higher yield of recombinant protein in homozygotes lines, these engineered tobacco plants could be useful for future EBOV mAb production.

## Author Contributions

C-YH, TL, and JX conceived and designed the experiments. YL, C-YH, CB, SN, and HR performed the experiments. C-YH, FK, TL, and JX analyzed the data. C-YH and JX wrote the article with contributions of all the authors.

## Conflict of Interest Statement

The authors declare that the research was conducted in the absence of any commercial or financial relationships that could be construed as a potential conflict of interest. The reviewer JF and handling Editor declared their shared affiliation at the time of review.

## Footnotes

1 http://www.who.int/mediacentre/factsheets/fs103/en/
