# Supplementary material for: An Effective Way of Producing Fully Assembled Antibody in Transgenic Tobacco Plants by Linking Heavy and Light Chains via a Self-Cleaving 2A Peptide
Source: Front Plant Sci. 2018 Sep 19;9:1379. doi: 10.3389/fpls.2018.01379 (PMC6156355; doi:10.3389/fpls.2018.01379)
Supplement: Supplementary file 1 [file Data_Sheet_1.PDF]

An effective way of producing fully assembled antibody in transgenic tobacco plants by linking heavy and light chains via a self-cleaving 2A peptide

Yuan Lin[1,2,¶] , Chiu-Yueh Hung[1,¶]·, Chayanika Bhattacharya[1], Starr Nichols[1], Hafsa Rahimuddin[1], Farooqahmed S. Kittur[1], TinChung Leung[3], Jiahua Xie[1,*]

¶: Equal contribution

[1]Department of Pharmaceutical Sciences, Biomanufacturing Research Institute & Technology Enterprise, North Carolina Central University, Durham, NC 27707, USA.
[2]School of Basic Medical Sciences, Ningxia Medical University, Yinchuan, China
[3]Department of Biological & Biomedical Sciences, Julius L. Chambers Biomedical Biotechnology Research Institute, North Carolina Central University, Durham, NC 27707, USA.

Jiahua Xie (✉) Tel.: (919) 530-6705; Fax: (919) 530-6600; E-mail: jxie@nccu.edu

SUPPLEMENTARY MATERIA

Supplemental Figure 1 | QRT-PCR of *HC* and *LC* in A92 and A93. (A) PCR amplification threshold cycles (Ct) at 8, 40, 200 and 1000 picogram (pg) of plasmid DNA of A93 and A92 were plotted. Data shown was the average of triplicate assays ± SD. (B) The cDNAs made from 40 ng of total RNA of seven independent transgenic plants from each A93 and A92 were PCR amplified. Their Ct values were used to calculate each equivalent amount of plasmid DNAs according to the standard curves in (A). Data plotted was the average of triplicate assays ± SD. All values were also listed in tables.

Supplemental Figure 2 | Immunoblotting analysis of crude protein extracts of A92 transgenic tobacco plants. Equal amounts of proteins were analyzed on SDS-PAGE under reducing conditions using anti-human IgG antibody. None was observed from A92 samples. Bottom panel shows equal protein loading by staining the blot with Amido Black 10B. IgG: 1 ng of human IgG. GUS: crude protein extracts of control plants carrying *GUS*. M: marker.

Supplemental Figure 3 | Quantification of EBOV mAbs in A92 and A93 leaves by ELISA using Protein A/G coated plates. The crude leaf extracts of six A92 and six A93 as well as the pooled T1 of A93 plants were used for ELISA. The amount of mAb was determined based on the human IgG standard curve. Data represent the amount of mAbs (ng) present in mg of total soluble protein (TSP). Data plotted were the average of three biological replicates ± SD.

Supplemental Figure 4 | T1 transgenic plants.

Supplemental Figure 5 | Determination of protein concentration isolated from A93 T1 plants. Eluted protein (15 µl) was compared to human IgG by immunoblotting analysis using anti-human IgG antibody. After elution some residual mAbs were still bound to beads when 0.25 µl of beads were examined by heating in 20 µl of LDS sample buffer.

(A)

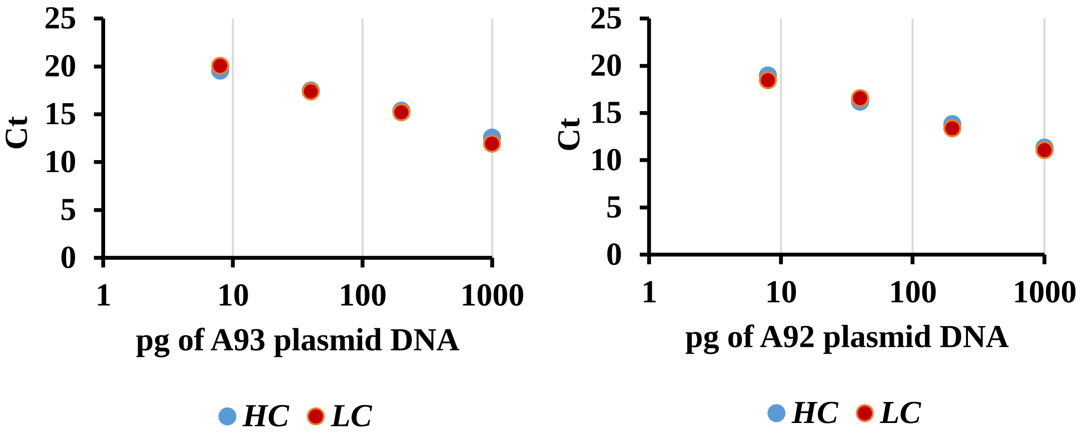

(B)

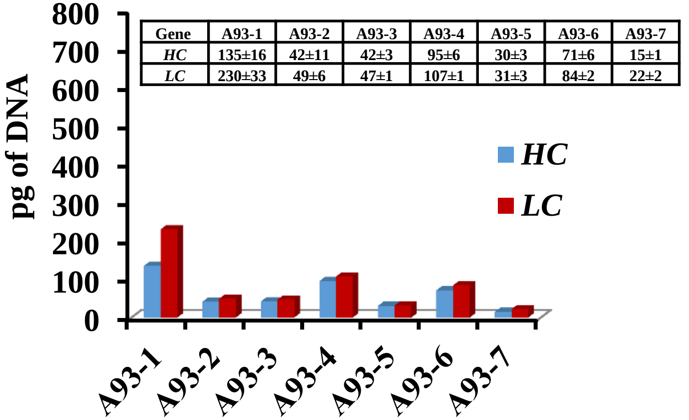

Gene	A93-1	A93-2	A93-3	A93-4	A93-5	A93-6	A93-7
HC	135±16	42±11	42±3	95±6	30±3	71±6	15±1
LC	230±33	49±6	47±1	107±1	31±3	84±2	22±2

Gene	A92-1	A92-2	A92-3	A92-4	A92-5	A92-6	A92-7
HC	288±12	297±105	773±97	639±31	143±21	335±10	20±1
LC	5±0	5±1	14±3	29±1	4±1	4±0	12±4

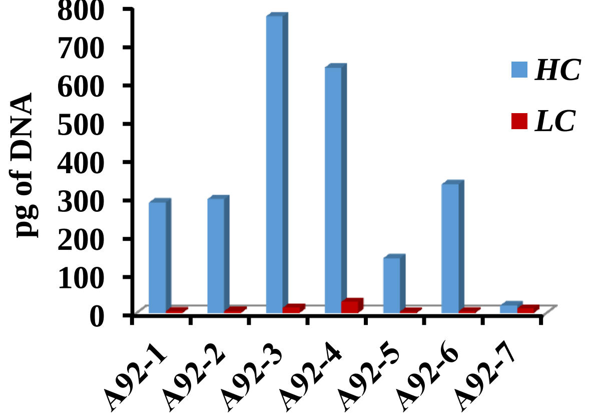

Supplemental Figure 1 | QRT-PCR of *HC* and *LC* in A92 and A93. (A) PCR amplification threshold cycles (Ct) at 8, 40, 200 and 1000 picogram (pg) of plasmid DNA of A93 and A92 were plotted. Data shown was the average of triplicate assays ± SD. (B) The cDNAs made from 40 ng of total RNA of seven independent transgenic plants from each A93 and A92 were PCR amplified. Their Ct values were used to calculate each equivalent amount of plasmid DNAs according to the standard curves in (A). Data plotted was the average of triplicate assays ± SD. All values were also listed in tables.

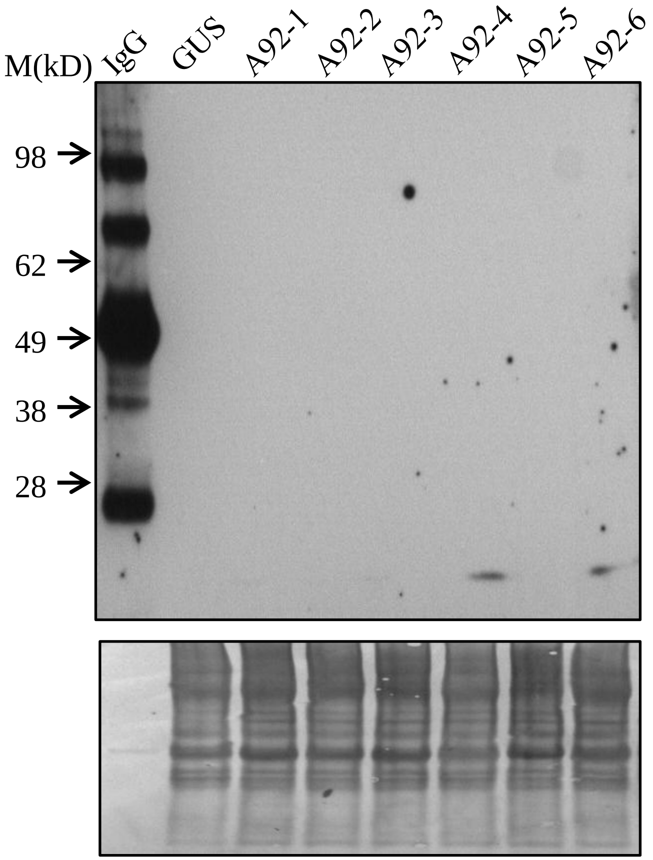

Supplemental Figure 2 | Immunoblotting analysis of crude protein extracts of A92 transgenic tobacco plants. Equal amounts of proteins were analyzed on SDS-PAGE under reducing conditions using anti-human IgG antibody. None was observed from A92 samples. Bottom panel shows equal protein loading by staining the blot with Amido Black 10B. IgG: 1 ng of human IgG. GUS: crude protein extracts of control plants carrying *GUS*. M: marker.

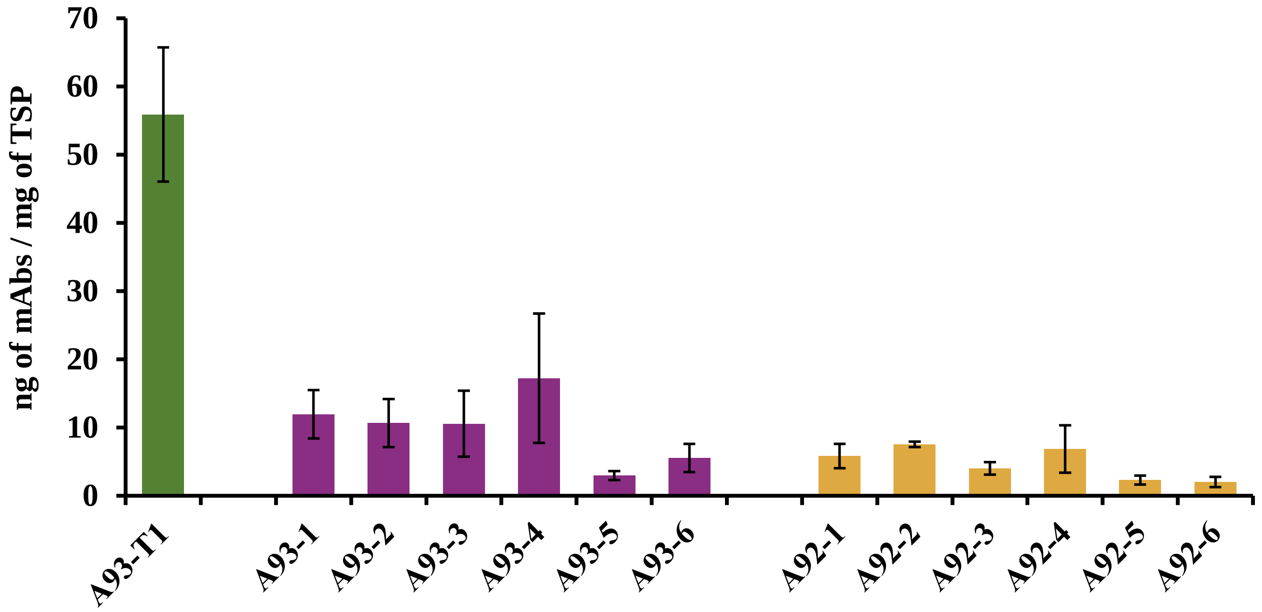

Supplemental Figure 3 | Quantification of EBOV mAbs in A92 and A93 leaves by ELISA using Protein A/G coated plates. The crude leaf extracts of six A92 and six A93 as well as the pooled T1 of A93 plants were used for ELISA. The amount of mAb was determined based on the human IgG standard curve. Data represent the amount of mAbs (ng) present in mg of total soluble protein (TSP). Data plotted were the average of three biological replicates ± SD.

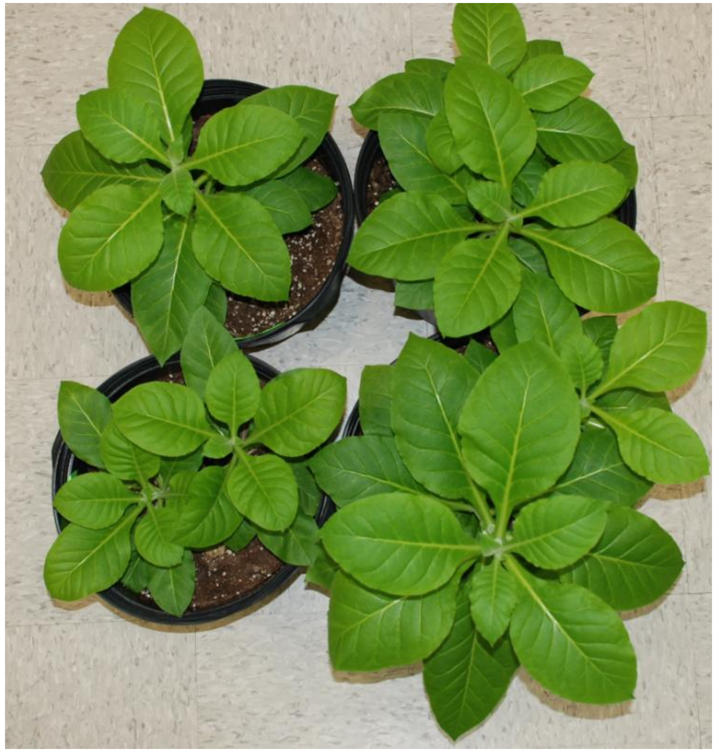

Supplemental Figure 4 | T1 transgenic plants.

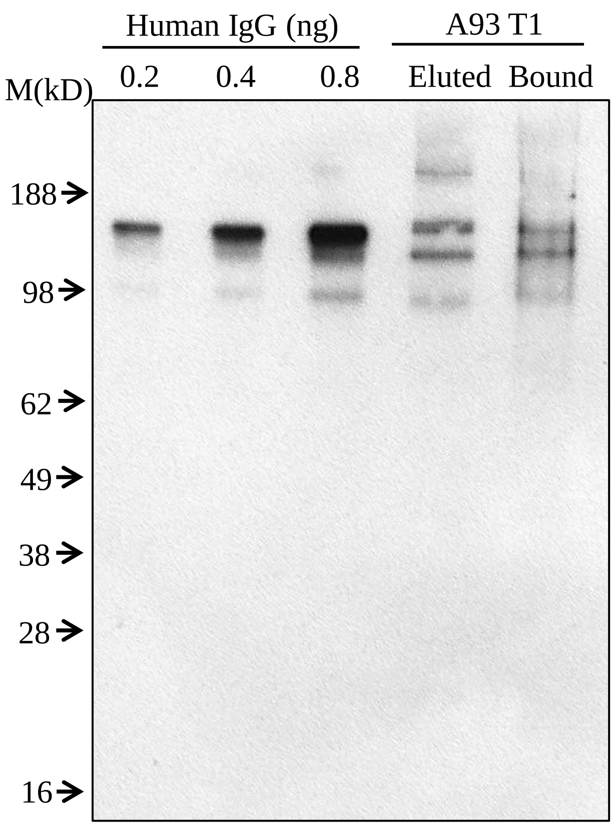

Supplemental Figure 5 | Determination of protein concentration isolated from A93 T1 plants. Eluted protein (15 µl) was compared to human IgG by immunoblotting analysis using anti-human IgG antibody. After elution some residual mAbs were still bound to beads when 0.25 µl of beads were examined by heating in 20 µl of LDS sample buffer.
